# DNA-damage and cell cycle arrest initiated anti-cancer potency of super tiny carbon dots on MCF7 cell line

**DOI:** 10.1038/s41598-020-70796-3

**Published:** 2020-08-17

**Authors:** Sinem Şimşek, Ayça Aktaş Şüküroğlu, Derya Yetkin, Belma Özbek, Dilek Battal, Rükan Genç

**Affiliations:** 1 Department of Chemical Engineering, Yıldız Technical University, 34210 Esenler, Istanbul, Turkey; 2 Department of Pharmaceutical Toxicology, Faculty of Pharmacy, Gazi University, 06330 Ankara, Turkey; 3 Advanced Technology Research and Application Center, Mersin University, 33343 Mersin, Turkey; 4 Department of Chemical Engineering, Faculty of Engineering, Mersin University, 33343 Yenişehir, Mersin, Turkey; 5 Department of Pharmaceutical Toxicology, Faculty of Pharmacy, Mersin University, 33169 Yenişehir, Mersin, Turkey; 6 Department of Pharmaceutical Toxicology, Faculty of Pharmacy, Near East University, 99138 Nicosia, Cyprus

**Keywords:** Medical research, Materials science, Nanoscience and technology

## Abstract

While carbon-based materials have spearheaded numerous breakthroughs in biomedicine, they also have procreated many logical concerns on their overall toxicity. Carbon dots (CDs) as a respectively new member have been extensively explored in nucleus directed delivery and bioimaging due to their intrinsic fluorescence properties coupled with their small size and surface properties. Although various in vitro/in vivo studies have shown that CDs are mostly biocompatible, sufficient information is lacking regarding genotoxicity of them and underlying mechanisms. This study aims to analyze the real-time cytotoxicity of super tiny CDs (2.05 ± 0.22 nm) on human breast cancer cells (MCF7) and human primary dermal fibroblast cell cultures (HDFa) by xCELLigence analysis system for further evaluating their genotoxicity and clastogenicity to evaluate the anti-tumor potential of CDs on breast adenocarcinoma. As combined with flow cytometry studies, comet assay and cytokinesis-block micronucleus assay suggest that the CDs can penetrate to the cell nuclei, interact with the genetic material, and explode DNA damage and G0/G1 phase arrest in cancer cells even at very low concentrations (0.025 ppm) which provide a strong foundation for the design of potentially promising CD-based functional nanomaterials for DNA-damage induced treatment in cancer therapy.

## Introduction

Functional nanomaterials that can better target cancer cells that will improve prevention and therapy could be accomplished by the combined efforts of nanotoxicologists, cancer biologists and nanobiomaterial scientists focused on toxicology and related cancer therapy. Engineering carbon-based nanomaterials and their applications are among the most dynamic fields in modern advanced materials science and engineering^1–3^. To date, various carbon nanomaterials such as carbon nanotubes^4,5^, fullerenes^6^, graphene^7,8^, graphene oxides^9^, carbon diamonds^10,11^, and carbon dots^12,13^ have been synthesized and reported by various researchers. Among these, CDs have become particularly interesting because of their unique physical and chemical properties such as thermal and electrical conductivity, high mechanical strength together with their unique optic and fluorescence features^14–16^. They have been used in bio-imaging, drug delivery, nucleus targeting, and labeling, photodynamic therapy, optoelectronics, solar cells, photocatalyst design, photodetectors, and many other biological and engineering fields^17–26^.

Several methods are available for the synthesis of CDs, namely, laser ablation^15^, electro-oxidation^27^ or oxidative acid treatment^12^; while nowadays, less laborsome and cheaper methods such as hydrothermal^28^ and thermal synthesis^24,29,30^, microwave-assisted hydrothermal synthesis^31^, ultrasound synthesis^32^, etc. are popularly exercised. CDs can be synthesized either from inorganic materials^19,33^ such as graphene^34^, carbon black^35^, and candle soot^12^ or biological carbon sources like food wastes, fruits, seeds and shells, plant extracts have been moving toward their application to biological sciences^36–38^.

Toxicity studies by various research groups have shown that CDs exhibit very low toxicity compared to heavy metal-based quantum dots when internalized by the cells^26,39–43^. In almost all of the cytotoxicity studies performed to date, CDs have been demonstrated to cause a negligible effect on cell viability at concentrations sufficient for drug delivery and bio-imaging. In the last 2 years, we have been focused on new types of CDs by tailoring the surface properties of them with the target of evaluating their potential in biological applications^16,44,45^. Very recently, utilizing different polymers as surface passivating agent and surface doping agent, we were able to show the immunostimulant and adjuvant-like effect of such CDs where no cytotoxic effect was observed^44,45^. CDs have been widely researched for nucleus-targeted delivery, nucleus labeling, photodynamic therapy, and optical monitoring of anticancer drugs^46–50^, however, so far there are few scientific reports in existence for addressing the genotoxic activities of CDs when they administrated on their own^51–56^. In a recent study, researchers showed the genotoxic responses of rat alveolar macrophages (NR8383) to amine-grafted graphene QDs which resulted in significant alterations in the expression of 2,898 genes after exposure for 24 h in which most of the down-regulated genes were reported the as they were responsive to “cell cycle”^57^. In a study on the use of graphene quantum dots (GQDs) as nucleus labeling of several cell lines (L929, HT-1080, MIA PaCa-2, HeLa, and MG-63 cells), researchers showed that internalization mechanism of the GQDs by healthy cells differ from tumor cells while GQDs showed to be entering into the nucleus regardless of the cell type. Moreover, the authors showed an altered number of L929 cells in the S phase as an indication of promoted cell proliferation in the presence of GQDs^50^. Yue et al.^25^ reported that ruthenium incorporated CDs with no apparent cytotoxicity have the concentration-dependent DNA photocleavage ability on HeLa cells upon the light irradiation (6.5 mW/cm^2^) showing the potential of CDs in imaging and photodynamic therapy (seeTable 2).

*Nerium oleander* (Oleander) is one of the most poisonous dwarf evergreen shrubs in the world^58^. Extracts from various parts of the plant show also anti-cancer^59,60^, anti-microbial^61^, anti-inflammatory^62^, anti-diabetic^63^, and neuroprotective activities^62^. Common ingredients of Oleander extract include polysaccharides containing rhamnose, galactose, arabinose, mannose, glucose, and galacturonic acid^64–67^. Other components and their concentrations may vary depending on the extraction method^68^. For instance, extracts of *N. oleander* leaves contain steroids, flavonoids, and terpenoids, etc.^67^. Our group lately presented the synthesis routes for Oleander based CDs using both thermal and microwave-based synthesis methodologies^69,70^ while we have shown that extract type (water or ethanol extraction) is one of the important parameters where the highest fluorescence and the lowest size was observed using water-based Oleander extract as a carbon source for CD synthesis.

The present study covers the effects of super tiny CDs on the cell viability of MCF7 tumor cells and normal HDFa cells together with the CD-induced differentiation in cell-cycle progression, genotoxicity, and clastogenicity on MCF7 cells. Our results suggest that CDs, alone or in combination with chemotherapeutics, may be exploited for the development of potentially promising functional nanomaterials for DNA-damage induced treatment in cancer therapy. They have the potential that could be extended to be used as new generation biolabeling and imaging agents as well. However, the possible influence of the cell cycle on cellular uptake of CDs and the mechanism of its effect on MCF7 cells needs further investigation.

## Materials and methods

### Plant material

*Nerium oleander* leaves collected from Esenler Region, Istanbul (41°01′37.70"N, 28°53′32.1"E) at 82 m May 2016 were booked in Izmir, Ege University Faculty of Pharmacy Herbarium (IZEF) with number 6056.

### Chemicals

Ethanol and Polyethylene Glycol (PEG 10000N), Dulbecco's Modified Eagle's medium (DMEM), trypan blue solution, Dulbecco’s Phosphate Buffered Saline (DPBS), agarose with normal melting point and low melting point, dimethyl sulphoxide, ethidium bromide, Triton X-100, phosphate-buffered saline tablets, potassium chloride (KCl), Giemsa, ethylenediaminetetraacetic acid disodium salt dihydrate (Na2-EDTA) and cytochalasin B (Cyt-B), the positive control for the genotoxicity assays, ethyl methanesulphonate (EMS) (CAS no. 62-50-0, lot 1338043) were obtained from Sigma-Aldrich (Steinheim, Germany). Trypsin Buffer, Tyrosine Inhibitor Buffer, RNase Buffer, Propidium Iodide Stain Solutions were purchased from Becton Dickinson (BD). Sodium chloride and sodium hydroxide were purchased from Merck Chemicals (Darmstadt, Germany), whereas Chromosome medium B was purchased from Biochrom AG (Berlin, Germany). Frosted microscope slides were obtained from Menzel GmbH (Braunschweig, Germany. Human breast adenocarcinoma cell line (MCF7) and the human primary dermal fibroblast cell cultures (HDFa) were obtained from ATCC with number HTB-22 and PCS-201-12, respectively. Slides were visualized for Comet Assay by fluorescence microscopy using an Olympus BX51 System equipped with a video camera CCD-4230.

### Equipment

ELMA TI-H 5 model ultrasonic bath was used during the extraction process. The thermal synthesis was conducted using a Neuve muffle furnace. Characterization studies of CDs were performed on a Shimadzu UV-1800 UV–Vis spectrophotometer, Agilent Cary Eclipse fluorescence spectrophotometer, Malvern Zeta Sizer Nano ZS, and Perkin Elmer frontier FT-IR. X-ray photoelectron spectroscopy (XPS) screening was performed using the Specs-Flex XPS spectrometer (Al Kα 1,486.7 eV). Morphology of CDs was monitored by a JEOL JEM-1400 series 120 kV Transmission Electron Microscope (TEM) and the FEI Tecnai G2 F30 HR-TEM at 300 kV. Particle core radius was calculated by measuring at least 100 individual particles using Image J program. Cell-seeding calculations were carried out with the Cedex XS analyzer (Innovatis Inc.). xCELLigence system (ACEA Biosciences Inc.) was used as a real-time cell sorter. BD FACSAria III flow cytometer (BD Biosciences, US) and BD CELLQuest Pro software (BD Biosciences, US) were used for cell-cycle analysis. Fluorescence imaging was performed by a fluorescence microscope (Olympus BX51) equipped with a CCD-4230 video camera.

### Preparing plant extracts

The fresh leaves of *N. oleander* were washed twice with distilled water and dried at incubator at 70 °C for 2 days. Dried leaves were then grinded to powders. The water extraction procedure was performed using ultra-pure water with the final concentration 12.5 g dry leaf/100 mL in ddH_2_O in an ultrasonic bath for 5 h at room temperature (RT). After extraction, clear extracts were obtained by centrifugation at 5,000 rpm for 15 min and stored at + 4 °C.

### Synthesis of CD using *N. oleander* leaf extract

1 mL aqueous extract was dispersed 1 g PEG solution pre-prepared in 2 mL of ddH_2_O/ethanol solution (1:1 v/v). The mixture then placed in a muffle furnace and left for the caramelization process for 45 min at 300 °C. Resulting brown solid was cooled down to RT and dissolved in 6 mL ddH_2_O. CDs were separated by three cycles of centrifugation at 13,500 rpm for 20 min. The supernatants were collected and dried in a vacuum oven at 60 °C for 1–2 days.

### Quantum yield of CDs

The quantum yield of the CD (diluted samples to obtain an absorbance value of less than 0.10) was determined using quinine sulfate in 0.1 M H_2_SO_4_ (quantum yield: 54%) as the standard sample following the procedure published previously^71^.

### In vitro cytotoxicity of CDs on MCF7 and the HDFa cell lines by xCELLigence

The xCELLigence system was used according to the manufacturer's instructions. The cell index (CI) was obtained by measuring the change in the electrical impedance in the presence and absence of cells in the wells. MCF7 and HDFa cell lines were inoculated keeping the cell number as 1 × 10^4^ cells/well and 3 × 10^4^ cells/well, respectively, on 16-well plates of the xCELLigence system. Afterward, cell growth in each well of e-plates was monitored every 15 min and analyzed with RTCA Software 1.2. After ~ 18–24 h of cell transplantation, cells in the ‘logarithmic growth phase’ were exposed to varying concentrations of CDs dispersed in DMSO (0.0025 ppm, 0.025 ppm, 0.25 ppm, 2.5 ppm, and 50 ppm), and monitored real-time for 72 h. The cells growing in the growth media were used as control while 50 ppm aqueous of *N. oleander* extract was used to compare with CDs. Experiments were carried out at least quadruplicate. All experimental steps were conducted under dark conditions to prevent additional light-induced cellular damages.

### In vitro genotoxicity of CDs on MCF7 cells by comet assay (SCGE)

Using the information obtained from the cytotoxicity studies, CD concentrations of 0.25, 2.5, and 50 ppm were selected for performing the alkaline comet assays. MCF7 cells in log-phase were plated in 96-well plates and incubated for 2 days. The negative control (Untreated cells that were grown in growth media, NC), and the positive control (Cells treated with 20 mM hydrogen peroxide) were inoculated in series. The comet assay was performed in alkaline conditions (pH > 13) as described previously^72^. Briefly, after cells were exposed to the CDs for 48 h and 72 h, the cells were collected and trypsinized. Centrifuged cells at 1,100 rpm for 5 min were counted and 1–3 × 10^4^ cells were resuspended in 75 μL molten 0.5% low-melting-point agarose at 37 °C. The resuspended cells in agarose were put onto dry microscope slides pre-coated with 1% normal-melting agarose, and the agar solidified by keeping at RT for 10 min. The slides were thereafter immersed in cold lysing solution (2.5 M NaCl, 100 mM EDTA, 10 mM Tris, 1% Triton X-100) for at least 1 h at 4 °C. Afterward, they were transferred to an electrophoresis tank containing freshly-made electrophoresis buffer (1 mM EDTA, 300 mM NaOH; pH > 13), where they were kept for 20 min at room temperature to allow DNA unwinding. Electrophoresis was performed in the same buffer at RT for 15 min at 24 V and 300 mA (0.8 V/cm). The slides were then neutralized thrice with 0.4 M Tris buffer (pH 7.5), air-dried, and fixed in ethanol. All preparative steps were conducted under dark conditions to prevent additional DNA damages. Slides stained with ethidium bromide (0.1 mg/mL, 1:4) were analyzed under a fluorescence microscope equipped with a CCD-4230 video camera.

### Slide scoring in comet assay

Comet images were analyzed following the method reported by Collins et al.^73^. The percentage of DNA in the comet tail from 100 cells per sample (duplicate, each with 50 cells/slide) was used as a measure of the amount of DNA damage. An intensity score from class 0 (undamaged) to class 4 (ultra-high damage) was assigned to each cell^74^. Observational blindness was employed, with the identity of the samples being withheld from the observer. Fifty cells per slide and two slides for each sample were examined to evaluate the DNA damage for each culture treated with CDs at different concentrations. The cells were classified by eye in the five categories based on the extent of DNA migration, undamaged (class 0), very little damage (class 1), moderate damage (class 2), high damage (class 3) ultrahigh damage (class 4). The ‘‘Arbitrary Unit (AU)’’ were used to express the extent of DNA damage and calculated using the following formula:

$$AU = \mathop \sum \limits_{i = 0}^{4} i x N_{i}$$

N_*i*_ = the number of scored cells in level *i*, *i* = the level of DNA damage (0, 1, 2, 3, 4).

### In vitro clastogenecity of CDs on MCF7 cells by cytokinesis-block micronucleus assay (CBMN)

The in vitro cytokinesis-block micronucleus assay (CBMN) was performed based on previously published procedures with minor modifications^75^. Cells were seeded at a density of 5 × 10^4^ cells per well in a T25 flask and treated with CDs in dispersion. After the exposure period of 48 h and 72 h, cells were washed twice with PBS and re-incubated for 38 h in fresh medium containing 3 μg/mL Cytochalasin B. Further, cells were harvested and suspended in ice-cold KCl for 50 s at room temperature. The cells were then fixed in Carnoy’s solution (1:3 mixtures of acetic acid and methanol), and several drops of formaldehyde were added to preserve the cytoplasm. Immediately after centrifugation at 1,500 rpm (1,000*g*) for 10 min, cells were fixed again in Carnoy’s solution. Finally, the cells were dropped onto clean microscopic slides, air-dried, and stained with Giemsa. A total of 1,000 binucleated cells for each sample were examined microscopically for micronuclei as previously described^76^. The levels of chromosomal damage were reported as the fold induction of micronuclei compared with the untreated control. CBMN results were accepted only when (1) they were separated from the main nuclei, but included within the corresponding cytoplasm, (2) they had a chromatin material similar to that of the main nuclei, (3) they were coplanar to the main nuclei, (iv)they were 1/16th to 1/3rd of the mean diameter of the main nuclei. In the CBMN study, toxicity was evaluated by classifying cells according to the number of nuclei^77^.

### Flow cytometry: cell cycle analysis of MCF7 cells treated with CDs

MCF7 breast cancer cells treated with CDs were analyzed by flow cytometry to determine the associated DNA index (DI) and to determine cell cycle phase distributions in these cells. 1 × 10^6^ cell/mL cells were plated in 6-well cell culture plates and treated with CDs at three different concentrations (0.25, 2.5, and 50 ppm) and incubated for 48 h and 72 h at 37 °C, 5% CO_2_. The cells were detached with trypsin, centrifuged (400*g*, 5 min.) and washed with PBS. Cells were then collected by centrifuge and treated with 250 µL trypsin buffer and vortexed. The obtained solution was mixed with Tyrosine Inhibitor and RNase Buffer (200 µL) and the mixture was incubated for 10 min. Further, 200 μL cold (2–8 °C) Propidium Iodide (PI) Stain Solution was added into each tube and incubated for an additional 10 min at 4 °C in dark with rapid stirring. The data were analyzed by Flow Cytometry Analysis Software. Values were expressed as fractions of cells in cell cycle phases (the mean ± standard error). Each experiment was performed three times.

### Statistical analysis

At least three independent experiments were carried out in triplicate for each evaluation. Data were expressed as the mean ± error (SE) and analyzed by repeated-measures ANOVA followed by the least significant difference post hoc test Bonferroni for the comet assay. An independent *t* test was used for the CBMN test. In all tests for comet assay and CBMN test differences were considered significant at *p* < 0.001 and *p* < 0.05 respectively. All the data analysis was carried out using software STATISTICA for comet assay and STATA MP/11 for the CBMN test.

## Results and discussion

### Characterization of physicochemical properties of CDs

**Figure 1:**
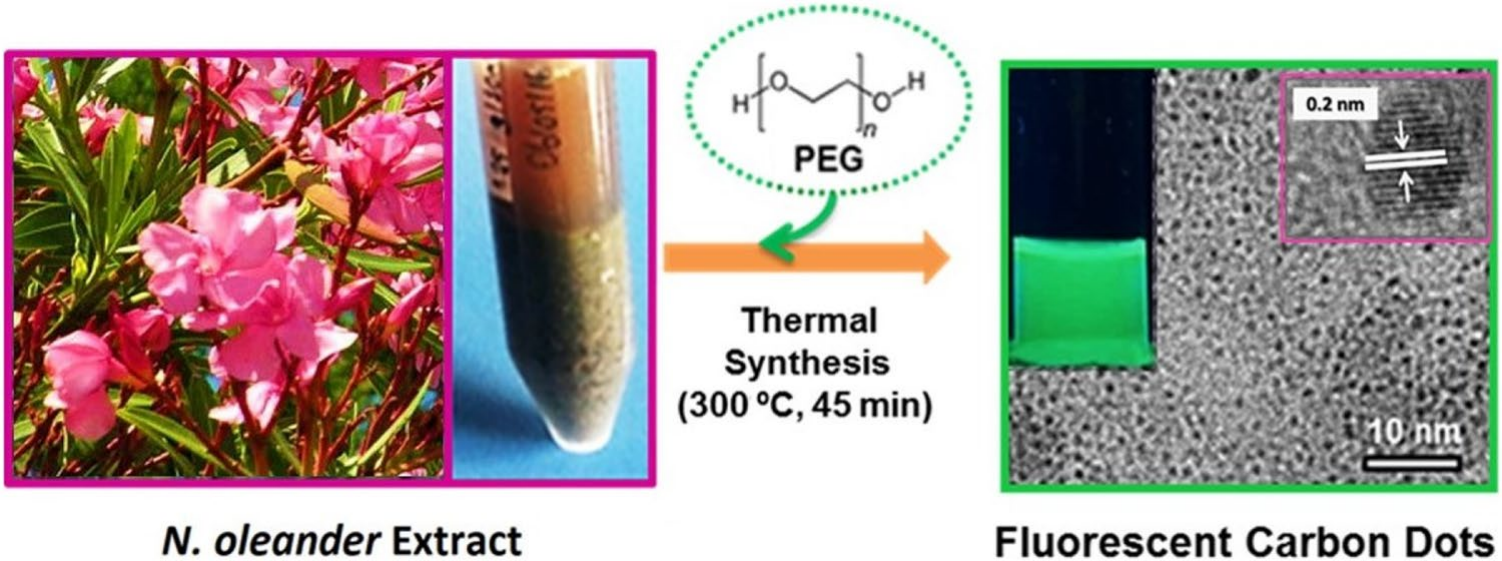
Schematics showing the experimental procedure of CD preparation from *N. Oleander* aqueous extracts and TEM and HR-TEM (Inset) images of as-synthesized CDs displaying a highly crystalline structure with a 0.21 nm lattice spacing that is attributed to the graphitic (*sp*^2^) carbon. Inset: photograph of the CDs emitting green colored fluorescence under a UV beam of 365 nm.

**Figure 2:**
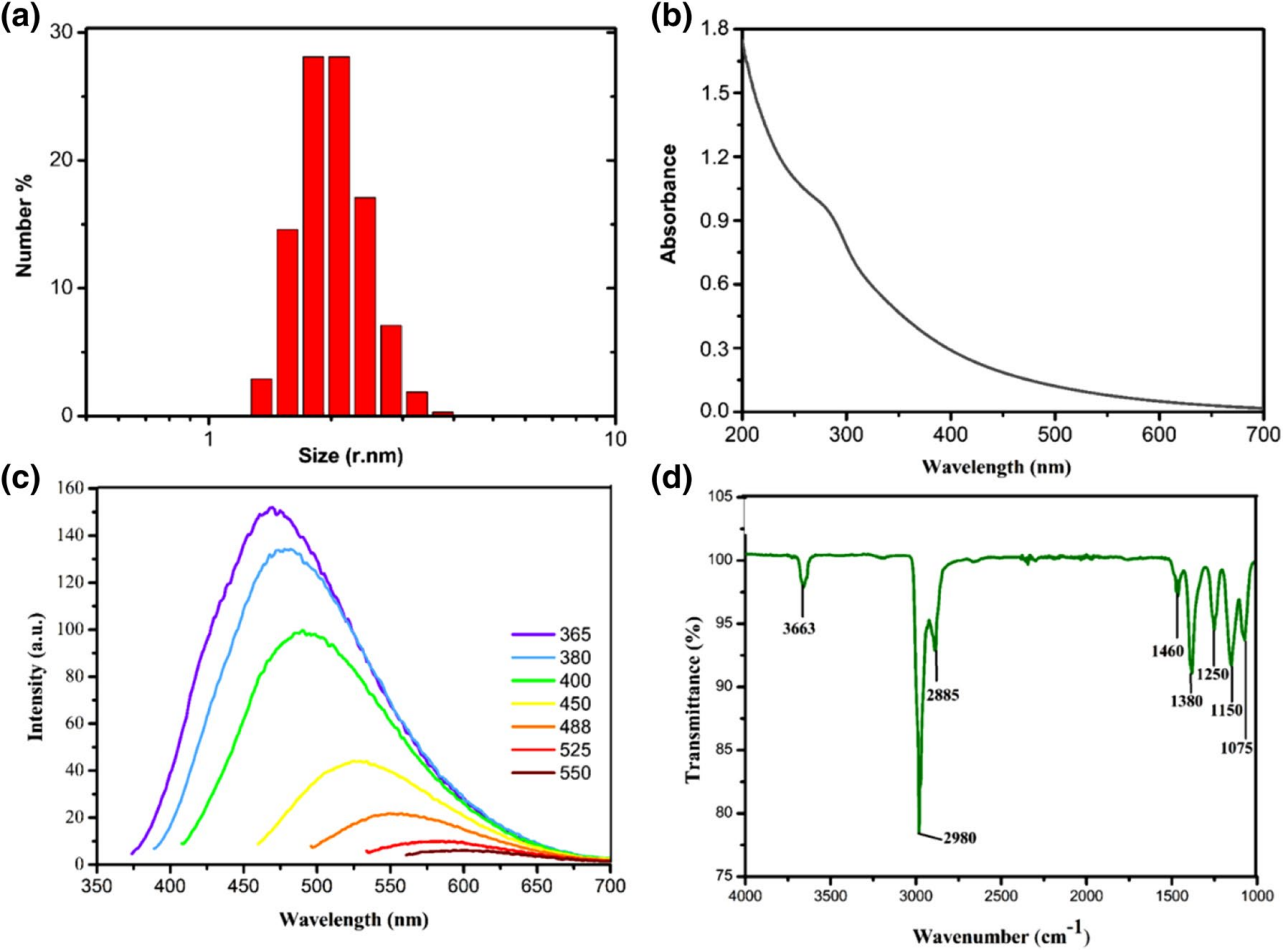
(**a**) Particle hydrodynamic size distribution of CDs measured by DLS, (**b**) UV visible spectrum, (**c**) excitation dependent emission spectra, and (**d**) FT-IR spectrum of CDs.

Many studies have attempted to elucidate the mechanisms of nanoparticle toxicity and distinguish between their bulk counterparts^78–80^. It is well-known that the toxicity of nanoparticles (NPs) was highly dependent on their physicochemical characteristics^81^. Table 1 shows some physical properties of as-synthesized and purified CDs (Fig. 1). PEG was used as a surface passivation agent which mainly acts as surface functionalization precursors for developing highly tunable photoluminescence properties by stabilizing the dangling bonds and controlling the surface functional groups and surface states^15,82,83^. Use of N. Oleander as carbon source and PEG as passivating agent revealed super tiny CDs with a hydrodynamic size (Rh) of 2.05 ± 0.22 nm (Fig. 2a) and core radius of 1.79 ± 0.33 nm measured by DLS and TEM imaging, respectively. The HR-TEM image (inset in Fig. 1) shows that the CDs displayed a highly crystalline structure with a 0.21 nm lattice spacing that is attributed to the graphitic (*sp*^2^) carbon^84^. Surface ζ-potential of − 23.5 ± 6.21 mV which could be the sign of a surface with the higher density of oxygen-rich groups^74,75^.

**Table 1:** Physicochemical properties of CDs.

Name	λ_max_ (nm)	Rh (nm)	r (nm)	ζ-Pot(mV)	σ (mS/cm)	m (µm cm/Vs)
CD	466	2.05 ± 0.22	1.79 ± 0.33	− 23.5 ± 6.21	0.05	− 1.84

λ_max_ is emission maxima at 365 nm excitation; Rh is the hydrodynamic radius, r is the core radius of CDs measured by TEM imaging, σ is electrical conductivity and m is electrical mobility.

CDs are exceptional carbon-based materials with superior optical properties that can be used in biomedical applications as they are trackable by fluorescence imaging. UV–Vis spectrum of CDs was presented in Fig. 2b. CDs showed a broad absorption peak located at around 245 nm which corresponds to a typical absorption of *sp*^2^ carbon network which was also supported by the HR-TEM imaging (Fig. 1)^16,85,86^. As can be seen in Fig. 2c, CDs showed the highest fluorescent emission at 466 nm upon excitation at 365 nm with the highest quantum yield of 7.12%. The fluorescence emission spectra of samples demonstrated an excitation-dependent feature originates from a combination of quantum confinement effects and the distribution of different emissive surface traps presented on the surface of CDs^82,87^.

FT-IR spectroscopy was carried out to characterize the surface properties of CDs. As depicted in Fig. 2d, CD showed a sharp peak around 3,600 cm^−1^ corresponds to free O–H groups present on the particle surface. The as-prepared CDs showed peaks belong to C–H stretches in methyl and methylene groups (2,800–3,000 cm^−1^)^88^. Peaks corresponding to C–O stretching located at 1,075–1,250 cm^−1^ might be associated with the partial oxidation of CD surfaces. Sharp peaks at 1,380 and 1,460 cm^−1^ were attributed to CH_2_ vibrations (Fig. 2d). Many oxygen-rich functional groups including C–O–C (1,150 cm^−1^), C–OH (1,250 cm^−1^) and C–OH stretching peak at 1,380 cm^−1^ could be indicative of a C–O–C asymmetric stretch or C–H bending arising from a methyl functional groups presenting on the CDs^35,49,89^.

**Figure 3:**
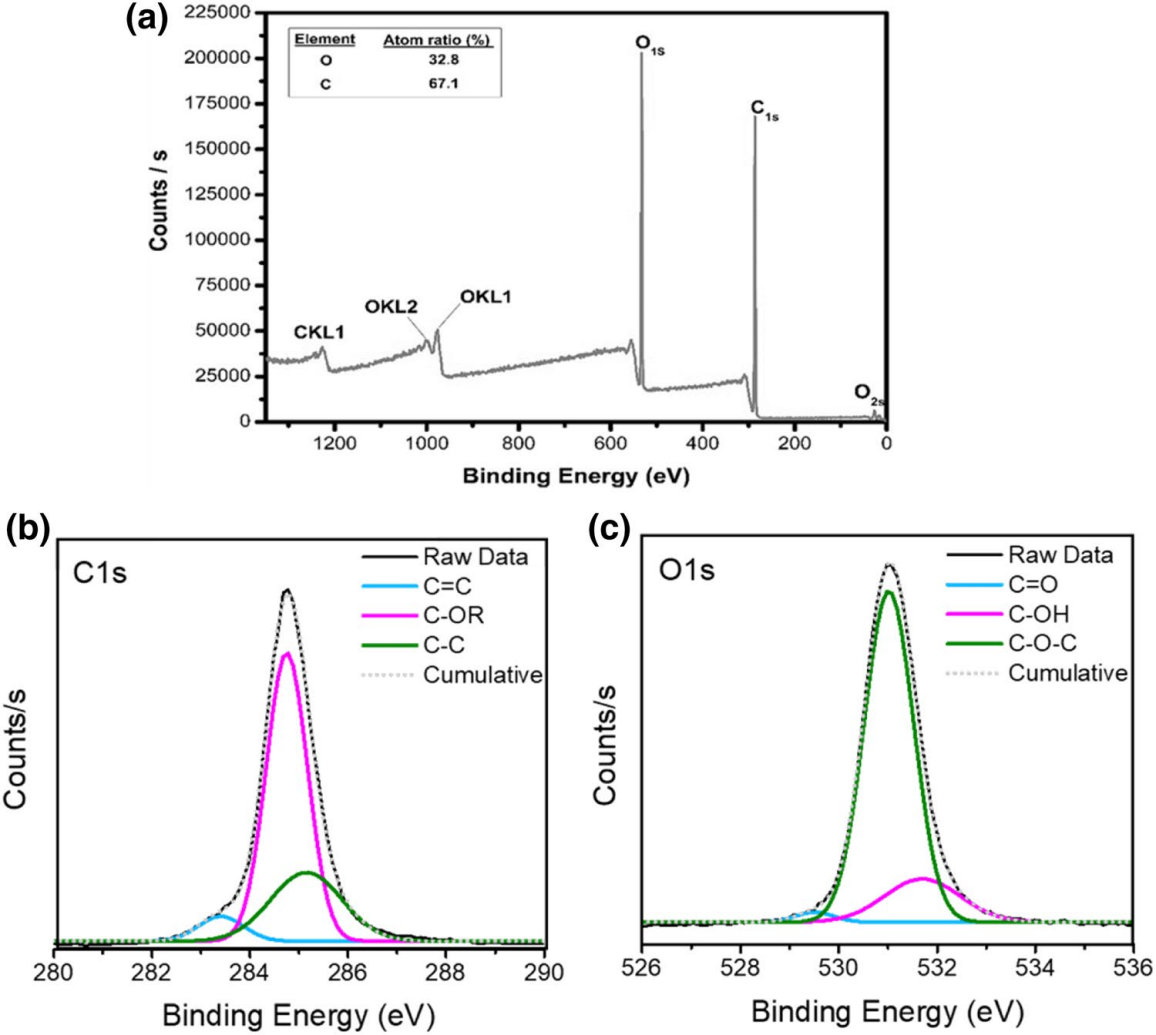
XPS spectra of the CDs. (**a**) Survey spectrum of the CDs with two major peaks of carbon and oxygen. XPS high-resolution survey spectra of (**b**) C1*s* and (c) O1*s* region of CDs.

Figure 3a depicts the XPS wide scan spectrum of the synthesized CDs. Two bands of the XPS survey spectrum at around 284.5 eV and 531.5 represented O1*s* and C1*s*, respectively, which indicates the atomic ratio of O/C is 32.8/67.2 as calculated from the survey spectrum. The high-resolution C1*s* XPS spectrum (Fig. 3b) was deconvoluted into three contributions at 283.4, 284.7, and 285.4 eV, which are associated with carbon in the states of *sp*^2^ C (C=C, C–C) and C–OR, respectively^90–92^. The deconvoluted O1*s* spectrum (Fig. 3c) had three components peaking at 529.4, 530.9, and 531.8 eV, which are due to the C=O, C–OH, and C–O–C groups, respectively^91,92^. XPS results support the UV–Vis spectrophotometry and FT-IR results that CDs are purely composed of C=C core and have a highly oxygenated and reactive surface.

### In vitro cytotoxicity of CDs on MCF7 and HDFa cells

Although MTT assay is one of the most used methods for the assessment of in vitro cytotoxicity of materials, there are reports on the interference of carbon-based nanomaterials with the colorimetric assays^93–95^. The xCELLigence is a technology that gives the possibility to measure the cellular growth in real-time by measuring the net adhesion of cells on a custom-designed gold electrode following the changes in electrical impedance. Thus, it gives more pre-sized information on the long-term screening of cell viability and prevents the material-dye interaction-based false responses^96^ The cytotoxicity of the CDs were conducted on MCF7 and HDFa cells. The normalized cell index (CI) is indicative of the level of adhesion and therefore associated with the viability of the cells.

**Figure 4:**
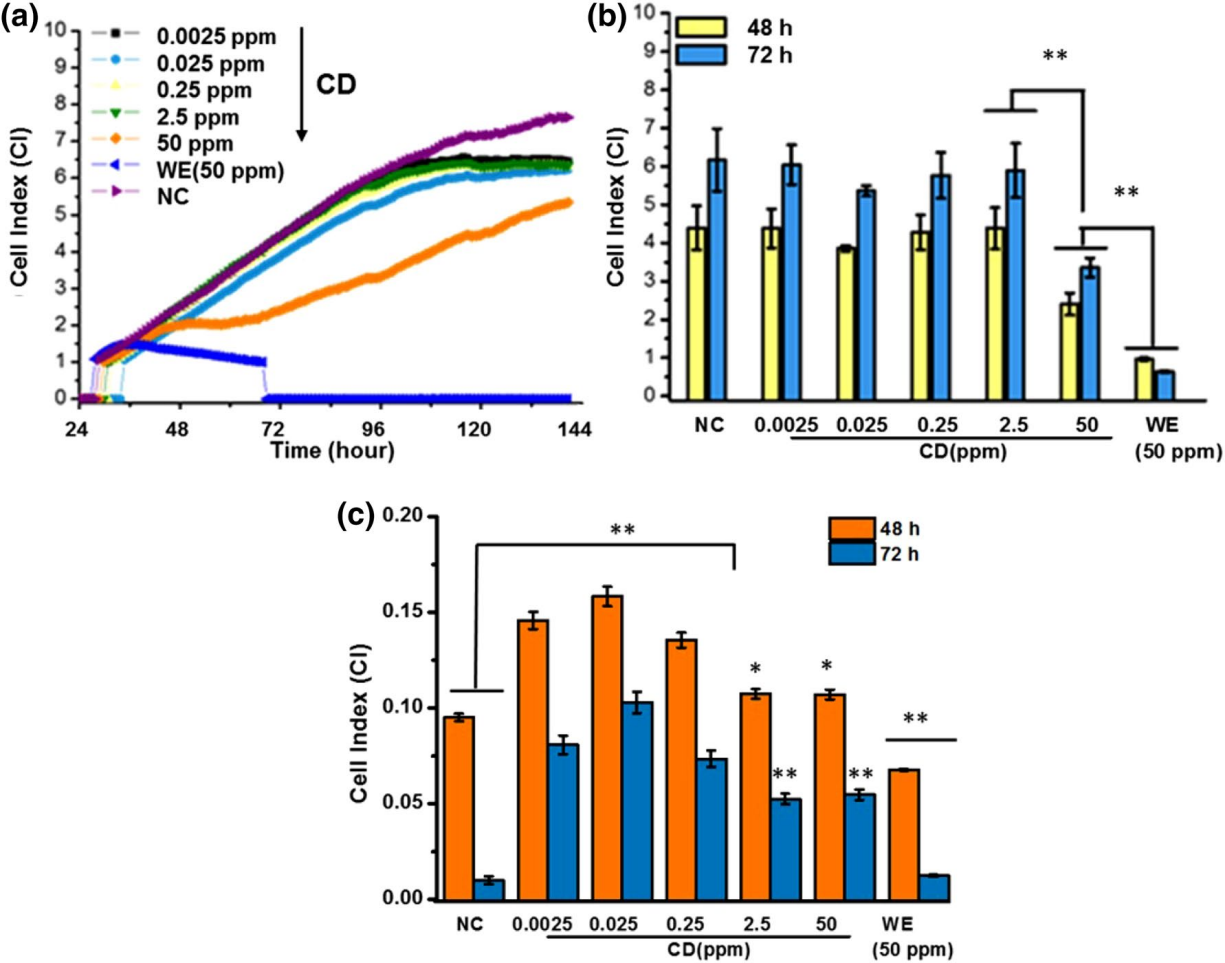
Time-dependent changes in cell index values and the average cell index values for (**a**), (**b**) MCF7 cells, and (**c**) HDFa cells at 48 h and 72 h after the treatment with varying concentrations of CDs as compared to the negative control (medium) and positive control (water extract of Oleandrin (50 ppm). Values represent mean ± SE, n = 3.

Figure 4a,b illustrates the normalized cell index of the MCF7 treated with varying concentrations of the CDs (0.0025–50 ppm) as compared to the PBS as the negative control. In all cases, cells were treated after 24 h following the seeding and the cell index was normalized at the point of treatment. The Oleander extract as control of the starting material resulted in cell death within a few hours after the treatment resulting in a sharp decrease in CI. After 24 h of treatment, CD at the highest concentration (50 ppm) revealed a significant decrease in cell growth as compared to the untreated controls. At lower concentrations, CDs did not show a similar growth trend as the untreated cells while no dose-dependent response was observed even after 72 h of treatment. These results agree well with experimental data taken from the literature that increased negative net charge and small size of CD increased the risk of cytotoxicity of them to tumor cells at high doses^49,97^. On the other hand, the story in the case of HDFa cells was different. As depicted in Fig. 4c, CDs did not show any cytotoxic effect at neither of the concentrations but also they significantly induced cell proliferation at CD concentrations of 0.25 ppm and below, while at 72 h of treatment with CDs even at the highest concentration resulted in increased cell proliferation which reveals that presence of CDs protected the cells from natural death. The CI value increased sixfold for the cells exposed to CDs at a concentration as low as 0.25 ppb as compared to the untreated cells. Oleandrin extract (50 ppm) was, as expected, showed to be cytotoxic for both of the cells regardless of the time of the exposure. Although the main reason is not clear yet, higher tolerability of non-cancerous cells, decreased or retarded cellular accumulation of CDs in healthy cells as compared to the cancerous cells have been highlighted in many recent reports to explain the enhancement of the cell proliferation of healthy cells after the CD exposure^50,81,98–100^. Yao et al.^101^ showed that CDs specifically interact with some cellular proteins of tumor cells and downregulate the level of some proteins and the activity of enzymes that are related to tumor cell invasion ability.

### Evaluation of genotoxicity and clastogenicity of CDs

**Figure 5:**
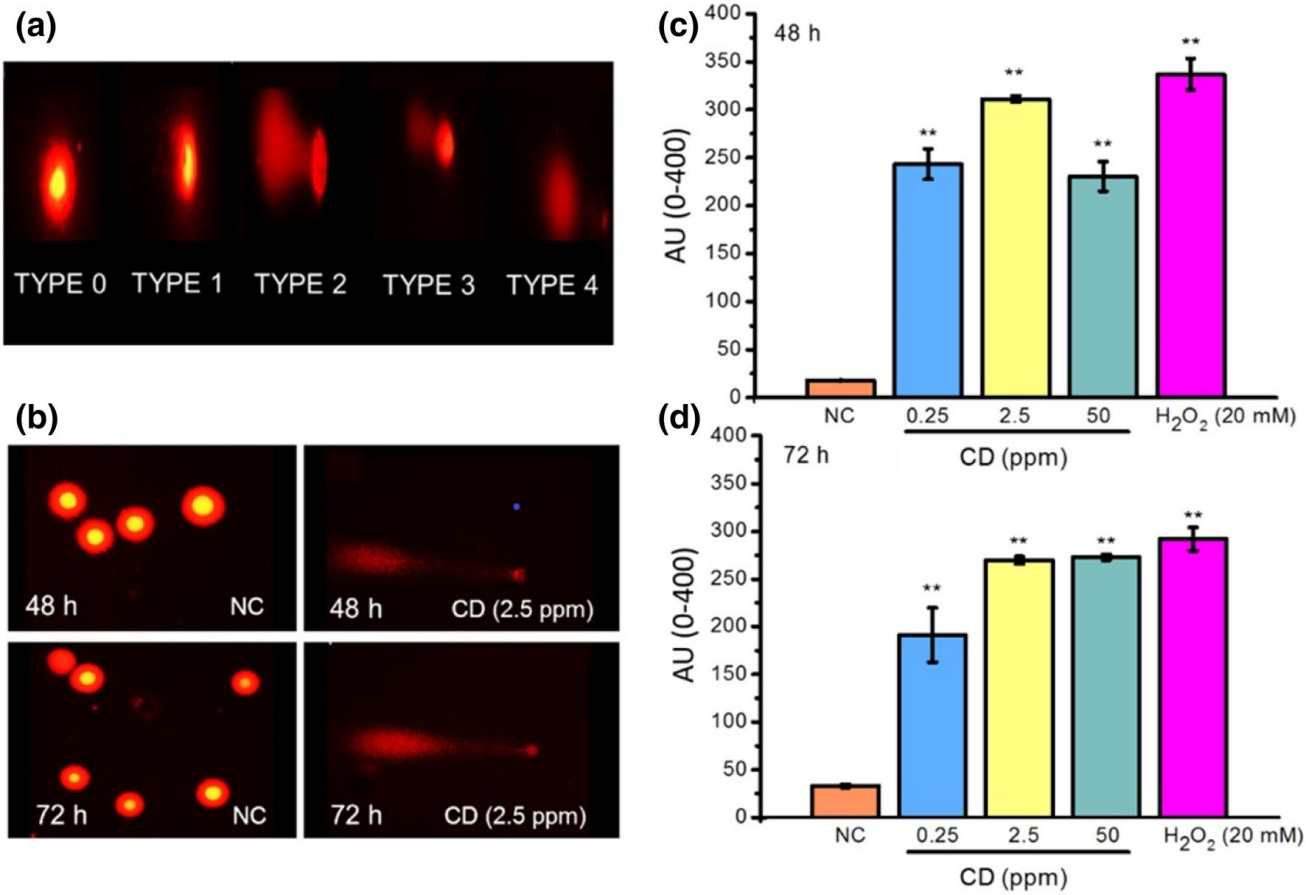
(**a**) Representation of the different comet classes in the alkaline comet assay where MCF7 cells were visually scored into four classes according to the tail length: Type 0: undamaged, with no tail, Type 1: with a tail shorter than the diameter of the head (nucleus), Type 2: with the tail as long as 1–2 × the diameter of the head, and Type3: with a tail longer than 2 × of the diameter of the head, Type 4: ultra-high damaged, with a longer tail length than the head diameter. (**b**) Representative images of non-treated control MCF7 cells and cells treated with 2.5 ppm CDs after 48 h and 72 h. DNA damage in MCF7 cells presented as arbitrary units (AU) measured by the alkaline Comet assay after treatment of cells with CDs for (**c**) 48 h and (**d**) 72 h. H202 (20 mM) was used as a positive control. (**) There is a statistically significant difference between positive control and CD-treated cells (*p* < 0.001).

The genotoxicity and clastogenicity of CDs on MCF7 cells were investigated by comet assay and CBMN, respectively. Figure 5 represents AU values indicating CD-induced DNA damage at various levels. AU values of negative control were calculated to be 18.50 ± 4.20 and 33.75 ± 6.24 after 48 and 72 h respectively. In contrast, the level of DNA damage jumped to 337.75 ± 9.81 (48 h) and 292.5 ± 16.36 AU (72 h) in positive control where cells were treated with H_2_O_2_ (20 mM). Increased levels of DNA damages induced by carbon dots occurred with respect to the negative control (Fig. 5). Even at the lowest CD concentration (0.25 ppm), AU value was elevated more than twofold as compared to the untreated cells. The difference between selected concentration groups was significant (*p* < 0.05). CD-based oxidative DNA damage can be correlated with several factors that strongly define the extent of CD-induced DNA-damage, such as the small size of these nanoparticles together with the surface charge and surface functional groups of them which alters the interaction of CDs with the cell^90,102–106^. Comet tail formation attributed to the DNA breaks or failed DNA repair mechanisms induced by oxidative stress. As supported by FT-IR, XPS, and ʐ-potential results, CD surface bearing a high amount of oxygenated functional groups could lead to the production of ROSs, and as a consequence, the oxidative stress may lead the genomic instability^97,107,108^. A recent study by Zhou et al.^90^ also indicated that modulating the oxygenated groups, most effectively the number of ketonic carboxyl groups, ROS production by carbon dots can also be controlled, the highest the oxygenated groups on the surface the highest the ROS production capability.

**Figure 6:**
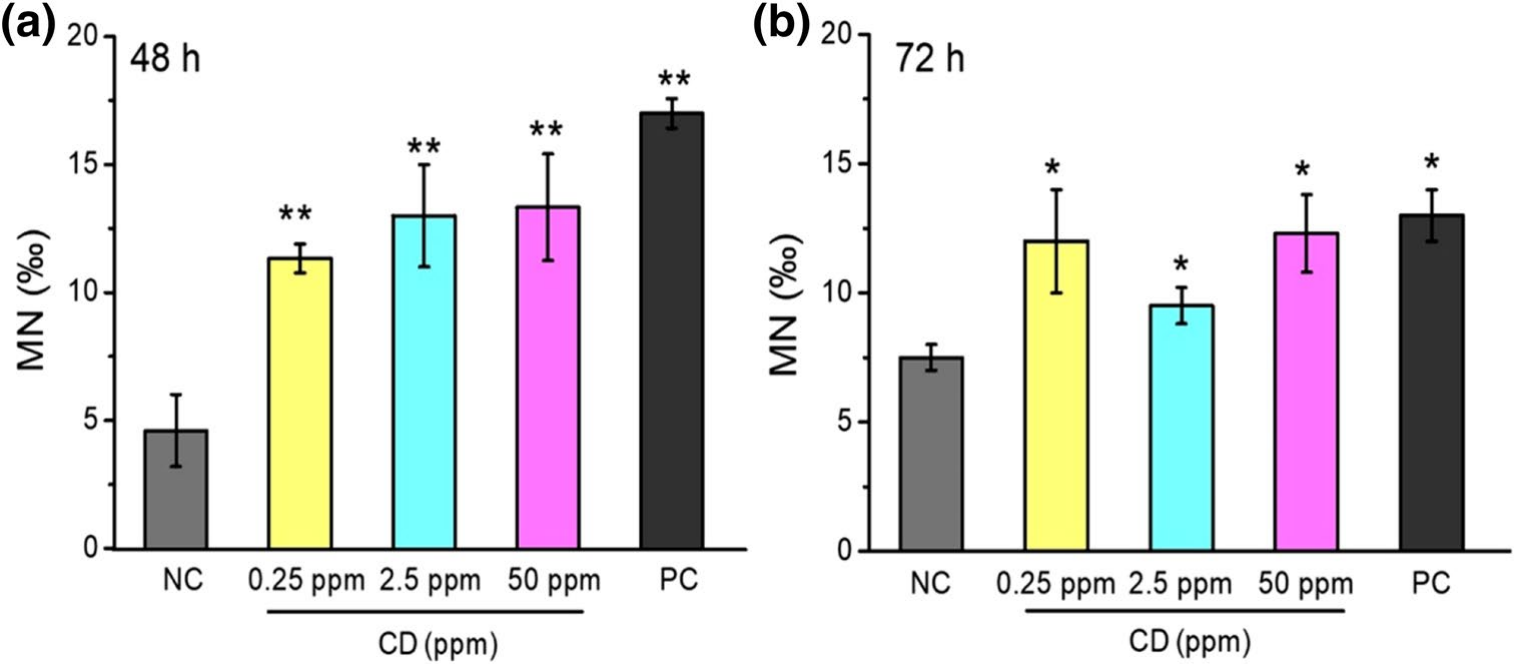
Changes in micronucleus frequencies in MCF7 cell lines treated with different concentrations of CD depending on the exposure time (**a**) 48 h and (**b**) 72 h. The statistically significant difference as compared to the negative control was shown as *p < 0.05 and **p < 0.01. Values represent mean ± SE, n = 3.

Clastogenic damage was also evaluated using CBMN for MCF7 cell lines (Fig. 6a,b) CDs contributed to a significant increase in DNA damage as compared to the negative control for all doses administered (0.25, 2.5 and 50 ppm) after their exposure for 48 h and 72 h (*p* < 0.01 and *p* < 0.05, respectively). A concentration-dependent enhancement of the level of clastogenic damage was observed at 48 h (Fig. 6a). After 72 h of exposure, the clastogenicity of CDs at 0.25 ppm was higher than CDs administrated at higher concentrations (Fig. 6b), and the micronucleus frequencies (MN%) decreased as compared to the lower concentrations after 48 h treatment. The difference between the MN value obtained for 0.25 ppm at two incubation times was significant (*p* < 0.05). The reduction overall in cellular damage at the 72nd hour can be explained by the induction of cellular damage repair mechanisms, which alters the protection of the tumor cell from further chemical attacks and reveal a subsequent recovery from existing ones at increased exposure time. The slight decrease in cell damage for higher CD concentrations can also be explained with the same phenomena. Decreased nanoparticle accumulation in tumor cells at increased exposure times have been also reported by many other researchers which might decrease the effect of CDs on tumor cells^100^. Another explanation of the occurring plateau might be the DNA damage which leads cells either to a necrotic death or irreversibly committing those to apoptotic cell death which eventually reduces the proportion of the highly damaged cells^109^.

### Influence of CDs on the cell cycle of MCF7 cells

**Figure 7:**
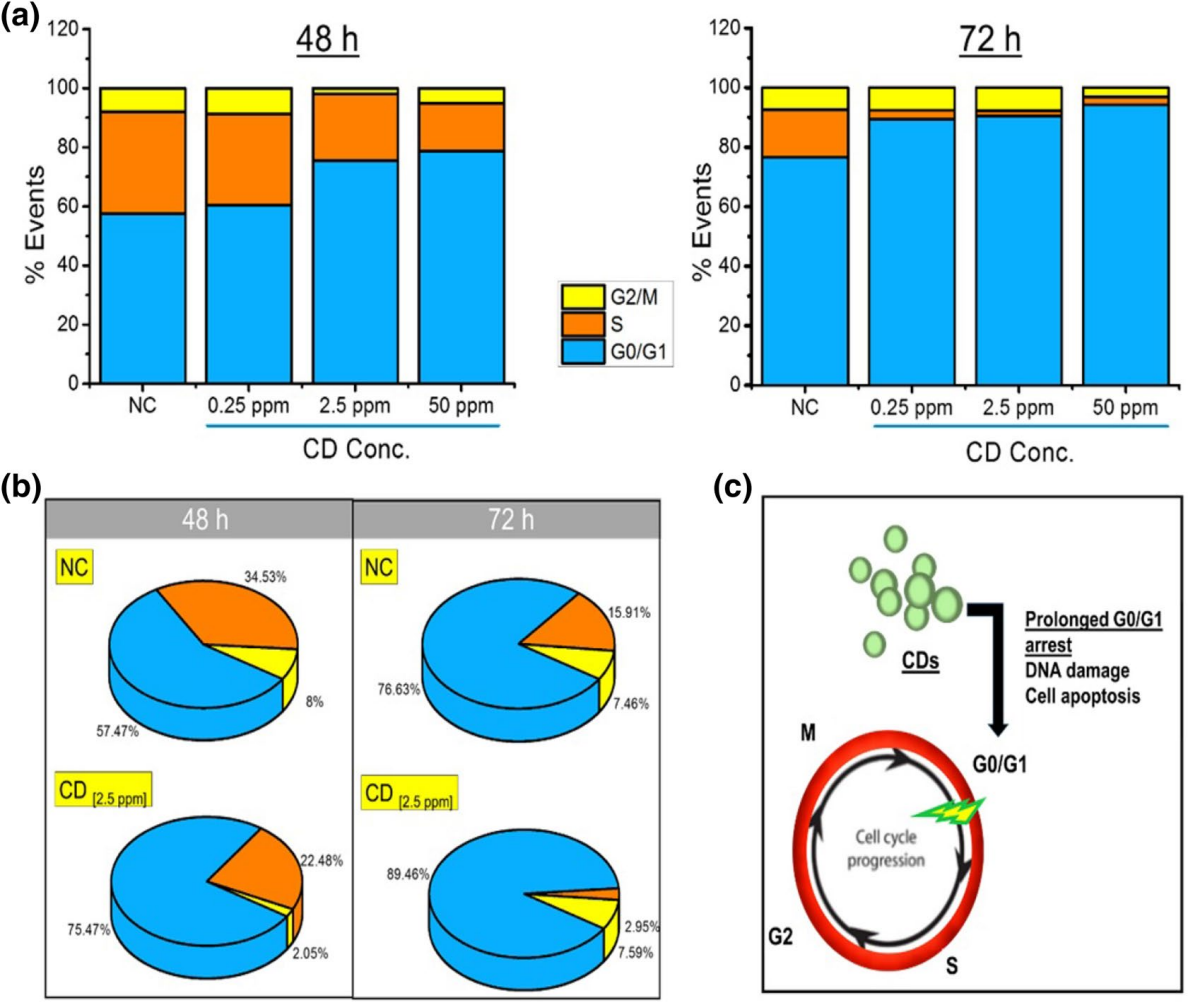
Statistical analysis of G0/G1, S, and G2/M populations in MCF cells (NC) and cells treated with (**a**) varying concentrations of CDs (0.25–50 ppm) for 48 h and 72 h. (**b**) Comparison of cell cycle phase profile of NC and CD (0.25 ppm) treated cells in different cellular phases. (**c**) Schematic representation of the possible effect of CDs in the cell cycle progression.

**Scheme 1.**
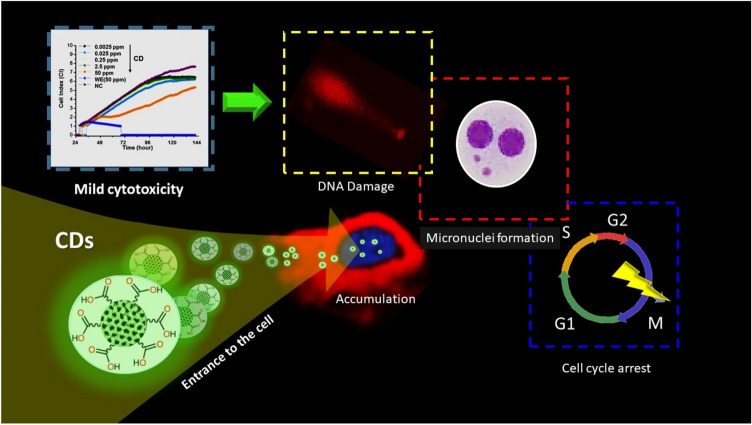
Overview of CD impact on Human Breast Cancer (MCF-7) Cell Line.

Considering the genotoxicity and clastogenicity induced by CDs, further analysis of the effects of CDs on the cell-cycle phases and apoptosis of MCF7 cells were performed. Cell proliferation is dependent on the cell-cycle progression, in which cells pass through the G0/G1 phase to the S phase and the G2/M phase. As depicted in Fig. 7a, after 48 h of treatment, 57.5%, 34.5% and 8.0% of untreated control cells were in the G0/G1, S and G2/M phases, respectively. Cells treated with CDs at even 0.25 ppm resulted in a decrease in the number of cells in the S phase (*p* < 0.05), and consequently a slight increase in the number of cells in the G0/G1 phases as compared to the control group. At increased CD concentrations, it has also been shown that induced cell cycle arrest in the G0/G1 phase is happening in a concentration-dependent manner, and the data were statistically significant as compared to the control group (*p* < 0.001). The effect of CDs on the cell cycle was shown to be time-dependent. As depicted in Fig. 6a,b, at 72 h, the changes in the cell cycle phases differentiated dramatically; 89.5%, 90.5% and 94.2% of MCF7 cells were in the G0/G1 phase respectively for cells treated with 0.25, 2.5 and 50 ppm CD (*p* < 0.005 for different concentrations of CDs), while only 76.6% of the control population was in the G0/G1 phase (*p* < 0.001). The significant differences (*p* < 0.001) in cell cycles for changing CD concentrations showing that prolonged delay in the G0/G1 phase is induced by CDs even at the lowest concentrations (0.25 ppm) which resulted in slower cell growth and delayed the entrance to S phase (Fig. 7a). As compared to untreated cells, CD (2.5 ppm) treated cells in S phase decreased by around 35% and 82% after incubation for 48 h and 72 h, respectively (Fig. 7b). Together with the genotoxicity and clastogenic analysis, the significant arrest of the cell population in G0/G1 phase, and the dramatic decrease in the population of cells in the S and G2/M phases support that CDs could trigger MCF7 cell apoptosis by inducing anomalies at G0/G1 phase by DNA damage and mutagenic stimulate (Fig. 7c). A comparison of the present study and previous literature is provided in Table 2. Most of the listed studies showed that negatively charged CDs with a size lower than 10 nm which can freely enter or interact with the nucleus while there are a couple of examples reporting nucleus targeting by larger sized CDs^47,92,100^, relatively few of them addressing the CD-induced anomalies in cell-cycle^49,108,110,111^. Our results suggest that CDs could have the potential as both drug carriers interacting with the cell-nucleus and therapeutic agents against tumor cells (Scheme 1). However, more studies should be done to handle this enormous therapeutic potential taking account the safety manners.

**Table 2:** Comparison of the results of the present study with earlier literature.

References		Carbon source		Synthesis method		Particle size		Application		Cytotoxicity
Li et. al.^111^		Ginger juice		Hydrothermal		8.2 ± 0.6 nm		Treatment of liver cancer		*Cell Type*: HepG2, human hepatocellular carcinoma cell line; MCF-10A, normal mammary epithelial cell line; FL83B, normal liver cell line; A549, human lung cancer cell line; MDA-MB-231, human breast cancer cell line Dose-dependent cytotoxicity, higher selectivity inhibition towards HepG2 cells Effective on HepG2 cell cycle (increase in SubG1 phase), did not cause significant differences in cell cycles for the other four non-cancerous cell lines
Kyung Yung et. al.^47^		Citric Acid and β-alanine		Microwave pyrolysis		2–3 nm		Cell nucleus Targeting		*Cell Type*: HeLa Cells No significant cytotoxicity, nuclear localization, Nucleus targeting, and imaging ability shown in vivo No genotoxicity evaluation
Havrdova et al.^49^		Candle soot		Oxidative acid treatment		4–7 nm		Cell nucleus Targeting and labeling		*Cell Type*: NIH/3T3, Standard mouse fibroblasts CD-Pri: low cytotoxicity, stimulated proliferation, evoked oxidative stress and induced abnormalities in the cell cycle (G2/M arrest), no entrance to the nucleus CD-PEG: low cytotoxicity, did not disrupt cellular morphology, toxic dose occurred at very high IC50 value and oxidative stress increased similarly like in the control. Did not cause any significant changes in the proportion of the cell cycle phases CD-PEI: cytotoxic, entering into the cell nucleus and inducing the largest changes in the G0/G1 phase of the cell cycle and also induced G2/M arrest
Periasamy et. al.^108^		Commercial CDs		-		< 50 nm		Cell cytotoxicity		*Cell Type*: hMSCs, human mesenchymal stem cells CNPs moderately reduce cell viability and cause chromatin condensation and DNA fragmentation, disrupt the expression of cell death genes Cell cycle progression of hMSCs was arrested slightly, the number of cells in G0/G1 increased at low concentrations of CNP exposure, cell cycle was arrested in the sub-G0/G1 phase in a dose-dependent manner
Kumawat et al.^50^		Grape seed extract		Microwave		50–60 nm		Nucleus Imaging, and Photoluminescent Sensing		*Cell Type*: L929, HT-1080, MIA PaCa-2, HeLa, and MG-63 cells The tendency to self-localize themselves into cell nucleus regardless of cell-type. No cytotoxicity and act as an enhancer in cell proliferation in L929 confirmed by in vitro wound scratch assay and cell cycle analysis. Enhanced the number of L929 cells in S-phase
Kalytchuk et. al.^110^		Citric acid and L-cysteine		Hydrothermal		3–6.5 nm		In vitro and in vivo luminescence lifetime thermometry		*Cell Type*: NIH/3T3, Standard mouse, fibroblasts; HeLa, human cervical cancer cells Low cytotoxicity, No significant effect on the cell cycle of HeLa cells, Dose-dependent G0/G1 arrest slightly on NIH/3T3 cells
Liu et al.^92^		Young Bearly Leaves		Hydrothermal		1.9 and 2.7 nm (in EtoH)		Cell nucleus Targeting and antiviral activity		*Cell Type*: PK-15 and HeLa cells No significant cytotoxicity, the neutral charged CDs (b-CDs) were localized in the cytoplasm and showed anti-viral activity, while the negatively charged ones (c-CDs) distributed through the whole cell and nuclear localization was also observed. Nucleus targeting and imaging ability of CDs have been shown in vitro No genotoxicity evaluation
Hill et al.^100^		Glucosamine-HCl and m-phenylenediamine		Microwave		2.42 ± 0.55 nm		LED-activated nucleus targeting and photothermal therapy		*Cell Type*: HDF and HeLa cells Less cytotoxicity on HDF than HeLa cells, nuclear localization in HeLa cell line, Nucleus targeting and imaging ability have been shown in vitro. CDs-based or LED induced cell death of cancer cells were not found to be associated with ROS production No genotoxicity evaluation
Zhang et al.^112^		Citric acid (CA), and propylene diamine (PDA)		Hydrothermal		5 nm		Cell nucleus labeling, cell-cycle imaging		*Cell Type*: HeLa-229 and HCerEPic No significant cytotoxicity on both cell lines. Permeability of cancer cells to CDs is higher than that of normal cells. N-CQDs were located in the nucleus with no fluorescence on the cytoplasm The majority of labeled HeLa cells were observed in interphase
Present study		*Nerium oleander*		Thermal		2.05 ± 0.22 nm		Anti-cancer therapy		*Cell Type*: MCF-7, human breast cancer cells, HDFa, human primer dermal fibroblast cells Dose-dependent cytotoxicity on MCF-7 cells, no cytotoxic effects on HDFa cells Genotoxicity, Clastogenicity and G0/G1 arrest on MCF-7 cells

## Conclusions

Although benign biocompatibility is attributed to carbon, systematic, and reliable biosafety assessment of carbon-based nanomaterials still needs to be conducted. Carbon dots serve as a useful and usable platform for a wide range of biological and biomedical applications including bio-imaging and nucleus targeted delivery/imaging. Herein, a systematic toxicity analysis of CDs produced from the extract of *N. oleander* via thermal synthesis method was conducted. In contradiction to many of the previous studies, we concluded that CDs have concentration and time-dependent cytotoxic potentials (at 50 ppm) over MCF7 cells while they comforted the proliferation of healthy HDFa cells even at highest CD concentrations. CDs caused severe DNA damage evident by the formation of COMET tail, micronuclei in MCF7 cells even at concentrations as low as 0.25 ppm. The interference of CDs with cell-cycle progression resulted in cell arrest in G0/G1 phases which showed that they can interact with genetic material and could trigger MCF7 cell apoptosis. Inducing Oxidative Stress Responses and interference with the cell cycle machinery could be due to the structure and surface properties of CDs while other mechanisms could also be involved. Although further studies are warranted to investigate the mutagenicity or carcinogenicity potency of CD in mammalian cells, this work shows evidence that CDs with super tiny size and high amount of oxygen on the surface can specifically affect the cellular function of tumor cells, and thus they have the potential to be used alone as an anti-cancer therapeutic material that can selectively target cancer cells by inducing series of DNA damage.
